# Efficacy of redifferentiation therapy in radioactive iodine refractory thyroid cancer: a systematic review and meta-analysis

**DOI:** 10.1007/s00259-026-08115-7

**Published:** 2026-08-06

**Authors:** Emilia Zampella, Valeria Cantoni, Roberta Green, Valeria Gaudieri, Federica Volpicelli, Leandra Piscopo, Mariano Scaglione, Alberto Cuocolo, Michele Klain

**Affiliations:** 1 Department of Advanced Biomedical Sciences, University of Naples Federico II, Via Pansini 5, 80131 Naples, Italy; 2 Department of Radiology, University of Sassari, Sassari, Italy; 3 IRCCS SYNLAB SDN, Naples, Italy

**Keywords:** Radioactive iodine, RAI-refractory thyroid cancer, Redifferentiation therapy, MAPK inhibitors, Selumetinib, Dabrafenib, Systematic review, Meta-analysis

## Abstract

**Purpose:**

To systematically review and meta-analyze the efficacy of redifferentiation therapy in patients with radioactive iodine (RAI)-refractory thyroid cancer.

**Methods:**

PubMed and Scopus databases were comprehensively searched up to January 2026 to identify clinical studies evaluating patients with RAI-refractory thyroid cancer who received targeted therapies to restore radioiodine uptake. Eligible studies reported either the rate of successful iodine re-uptake or treatment response evaluated according to RECIST 1.1 criteria. The primary endpoint was the pooled rate of successful redifferentiation, while secondary endpoints were objective response rate (ORR) and disease control rate (DCR), each stratified by redifferentiation status.

**Results:**

Fifteen studies comprising 234 patients (cohort sizes: 3–33) were included. The pooled rate of successful redifferentiation was 51% (95% confidence interval, CI: 30%-71%), with no significant publication bias. Treatment response according to RECIST 1.1 was evaluated in 11 studies (93 patients). The pooled ORR was 28% (95% CI: 13%-44%) in redifferentiated patients compared to 8% (95% CI: 0%-44%) in non-redifferentiated individuals (*P* = 0.33). The pooled DCR was significantly higher, reaching 93% (95% CI: 79%-100%) in redifferentiated patients compared to 54% (95% CI: 15%-91%) in non-redifferentiated patients (*P* = 0.04).

**Conclusions:**

In patients with RAI-refractory thyroid tumors, redifferentiation therapy using mitogen-activated protein kinase inhibitors successfully restores radioiodine avidity in approximately half of the cases. Although short-term disease control rates are promising, statistically significant prognostic differences and long-term survival benefits remain to be demonstrated in larger, standardized trials with extended follow-up.

**Supplementary Information:**

The online version contains supplementary material available at https://doi.org/10.1007/s00259-026-08115-7.

## Introduction

Radioactive iodine (RAI) therapy with ^131^I is a well-established treatment for patients with differentiated thyroid cancer (DTC) [1–3]. According to the 2025 American Thyroid Association (ATA) guidelines, RAI therapy is strongly recommended for patients at high risk of recurrence [3]. A significant benefit of radioiodine administration has also been demonstrated in patients with metastases, particularly those with lung lesions [4]. However, adequate radioiodine uptake in target tissues is mandatory to achieve a successful therapeutic response. Patients with metastatic DTC may exhibit intrinsic or acquired radioiodine resistance during the course of the disease. Refractoriness to RAI therapy is defined as the absence of ^131^I uptake in known structural disease, or as disease progression less than 6 months after RAI treatment [3]. Several factors are associated with lower RAI avidity in metastatic tissue, including advanced patient age, large tumor size, and specific histological types [5]. Moreover, mutational events involving the mitogen-activated protein kinase (MAPK) pathway indicate aggressive tumor features and are frequently associated with RAI refractoriness and poorer outcomes [6, 7]. Consequently, patients with RAI-refractory disease should not receive further empiric radioiodine treatments, and alternative strategies must be considered. Redifferentiation therapy using kinase inhibitors has been proposed to restore iodine uptake in metastatic lesions [8]. Although several studies show encouraging data regarding redifferentiation rates, the exact impact of this strategy on long-term clinical outcomes remains unclear. Therefore, this systematic review and meta-analysis aim to investigate the efficacy of redifferentiation therapy in patients with RAI-refractory thyroid cancer.

## Methods

This systematic review and meta-analysis followed the Preferred Reporting Items for Systematic Reviews and Meta-analyses (PRISMA) statement (PRISMA Checklist available in the Supplementary Material) [9] and registered as 420261399909 in the PROSPERO database (University of York, UK; http://www.crd.york.ac.uk/PROSPERO).

### Search strategy

A comprehensive literature search was performed in PubMed and Scopus databases to identify English-language articles published up to January 2026, with no restrictions on the earliest publication date. The search was restricted to human adult populations using the following Boolean search string: ("thyroid cancer"[All Fields]) AND ("radioiodine refractory"[All Fields] OR "radioactive iodine refractory"[All Fields] OR "RAI refractory"[All Fields]) AND ("redifferentiation therapy"[All Fields]) AND ("prognosis"[All Fields] OR "follow-up"[All Fields] OR "response to therapy"[MeSH Terms] OR "clinical outcome"[All Fields]).

### Study selection

Two reviewers (V.C. and R.G.) independently screened the titles and abstracts of potentially relevant studies. Disagreements were resolved by consensus. Observational clinical trials, prospective studies, and retrospective studies were considered eligible. Full-text articles of the selected reports were retrieved and independently evaluated by the same two reviewers based on the eligibility criteria. The reference lists of the retrieved articles were manually screened to identify additional relevant citations.

### Study eligibility and data extraction

Each study was initially categorized by journal, authors, and year of publication. We included studies evaluating patients aged ≥ 18 years with RAI-refractory thyroid cancer who received targeted therapies to restore radioiodine uptake. Eligible studies were required to report at least one of the following outcomes: 1) the percentage of patients in whom targeted therapy successfully restored iodine uptake in the index tumor, or 2) treatment response evaluated according to RECIST 1.1 criteria [10] as complete response (CR), partial response (PR), stable disease (SD), or progressive disease (PD). For studies reporting data on distinct patient populations, each cohort was extracted and analyzed separately. Editorials, abstracts, animal studies, conference presentations, non-English publications, and studies not focused on the specific topic of interest were excluded.

### Assessment of the methodological quality of studies

The methodological quality and risk of bias of the included studies were assessed for methodological quality using the Joanna Briggs Institute (JBI) Critical Appraisal Checklist for Studies Reporting Prevalence Data [11], focusing on the 8 relevant domains: condition measurement reliability, condition measurement validity, data analysis, participant recruitment, sample size, statistical analysis, study subjects/setting, and target population. Two reviewers (V.C. and R.G.) independently performed the quality assessment, resolving discrepancies through consensus. A final rating of "yes" (+), "no" (−), or "unclear" (+/−) was assigned to each item. Studies with "yes" for all items were classified as having a low risk of bias; studies with one or more "no" or "unclear" responses were classified as moderate risk; studies with entirely "no" responses were categorized as high risk of bias.

### Statistical analysis

Continuous variables were expressed as mean ± standard deviation, and categorical data as percentages. The primary endpoint was the percentage of patients in whom targeted therapy successfully induced radioiodine re-uptake. Secondary endpoints included the objective response rate (ORR), defined as the percentage of patients achieving CR or PR, and the disease control rate (DCR), defined as the percentage of patients achieving CR, PR, or SD, both stratified by redifferentiation status.

The meta *esize* command in Stata was used to perform meta-analyses of proportions to derive pooled estimates. To handle proportions, close to the margins, within-study variability was modeled using the exact binomial distribution, and the Freeman–Tukey double-arcsine transformation was applied to stabilize variances. All analyses were conducted using logit-transformed proportions as the effect size statistic, weighted by the inverse of the variance, and back-transformed to proportions for final presentation.

Inter-study heterogeneity was evaluated using the *I*^*2*^ statistic, where values of 25%, 50%, and 75% represented low, moderate, and high heterogeneity, respectively [12]. In accordance with the Cochrane Handbook, an *I*^*2*^ > 50% indicated substantial heterogeneity [13], necessitating the use of a random-effects model [14]. Publication bias was assessed graphically using funnel plots and formally evaluated via Egger’s regression asymmetry test [15, 16]. A leave-one-out sensitivity analysis was performed to determine the influence of individual studies on the overall effect size and heterogeneity. Meta-regression analysis was conducted to examine whether study-level covariates, including age, female percentage, papillary thyroid carcinoma (PTC) percentage, and *BRAF*/*RAS* mutation rates, were associated with the pooled prevalence of successful redifferentiation. Subgroup analyses were conducted according to type of MAPK inhibitor (BRAF inhibitor vs. MEK inhibitor vs. multi-kinase inhibitor) and criteria for defining successful redifferentation (WBS interpretation vs PET/CT dosimetry).

All analyses were performed using Stata version 18.0 (College Station, TX). Two-sided P-values < 0.05 were considered statistically significant.

## Results

### Study selection

**Fig. 1:**
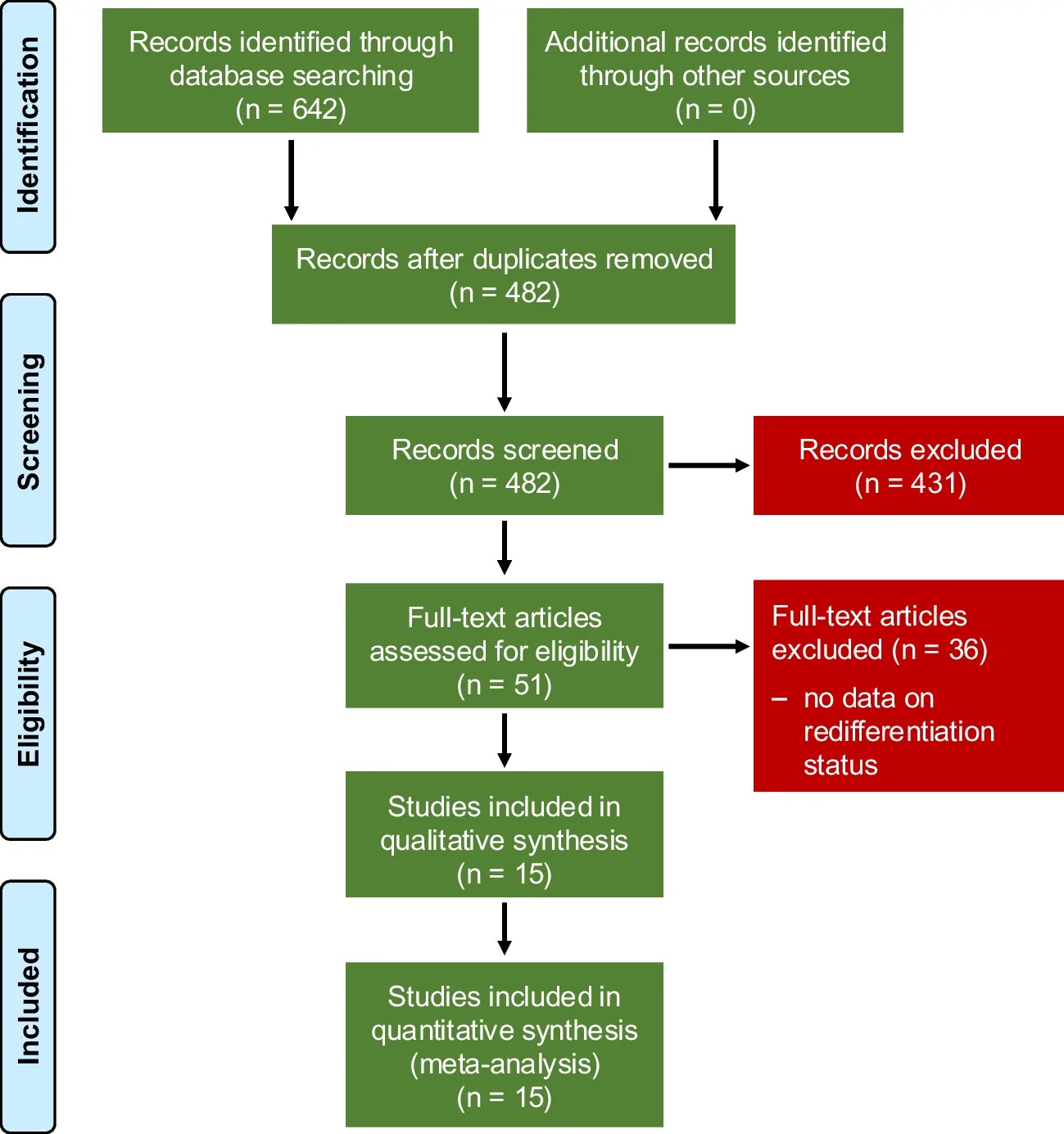
PRISMA flowchart illustrating the study selection process

The complete literature search process is depicted in Fig. 1. A total of 642 potentially eligible citations were initially identified through database searching. After the removal of duplicates, 482 unique records remained. Following the initial screening of titles and abstracts, the reviewers excluded 431 citations because they were deemed non-relevant or non-pertinent. The full texts of the remaining 51 articles were reviewed by each investigator to determine eligibility; disagreements were resolved by consensus. Thirty-six articles were excluded after full-text evaluation due to a lack of data on redifferentiation status. Consequently, the remaining 15 articles [17–31], comprising a total of 234 patients, were included in the meta-analysis.

### Characteristics of the included studies

Table 1 reports the study design along with the demographic and clinical characteristics of the pooled patient population. Ten studies were conducted prospectively [17–22, 25, 27, 28, 30] and 5 retrospectively [23, 24, 26, 29, 31]. Sample sizes ranged from 3 to 33 patients, and the median age ranged from 48 to 72 years. Regarding histological subtypes, 144 patients had PTC, 66 had follicular thyroid carcinoma (FTC), and 24 had poorly differentiated thyroid cancer. Furthermore, a genetic mutation involving the MAPK pathway was reported in 184 patients, whereas 24 patients exhibited a wild-type profile. Table 2 summarizes the characteristics of the patients included in this meta-analysis alongside the specific outcome measures of the selected studies.

**Table 1:** Study design and demographic/clinical characteristics of the pooled patient population

Study	Design	Patients (n)	Age (y)	Histology (*n*)	Mutation Profile (*n*)
Ho et al. [17]	Prospective	20	61 (44–77)	PTC (13), PDTC (7)	*BRAF* (9), *RAS* (5), Other (3), WT (3)
Rothenberg et al. [18]	Prospective	10	66 (61–84)	PTC (10)	*BRAF* (10)
Dunn et al. [19]	Prospective	12	68 (43–72)	PTC (9), PDTC (3)	*BRAF* (12)
Tchekmedyian et al. [20]	Prospective	7	67 (50–83)	PTC (7)	*BRAF* (7)
Dotinga et al. [21]	Prospective	9	63 (55–79)	PTC (3), FTC (6)	*RAS* (3), Other (6)
Wadsley et al. [22]	Prospective	28	66 (45–83)	PTC (11), FTC (17)	NA
Toro-Tobon et al. [23]	Retrospective	33	65 (55–69)	PTC (19), FTC (11), PDTC (3)	*BRAF* (18), *RAS* (12), Other (3)
Groussin et al. [24]	Retrospective	3	NA	PTC (2), FTC (1)	NA
Hoftijzer et al. [25]	Prospective	32	65 (53–82)	PTC (16), FTC (16)	*BRAF* (10), *RAS* (3), Other (19)
Jaber et al. [26]	Retrospective	13	56 (45–75)	PTC (10), FTC (1), PDTC (2)	*BRAF* (9), *RAS* (3), WT (1)
Leboulleux et al. [27]	Prospective	11	66 (22–77)	PTC (4), FTC (2), PDTC (5)	*RAS* (11)
Leboulleux et al. [28]	Prospective	24	67 (51–81)	PTC (24)	*BRAF* (24)
Pitoia et al. [29]	Retrospective	4	48 (28–76)	PTC (4)	*BRAF* (4)
Weber et al. [30]	Prospective	20	65 (48–83)	PTC (10), FTC (7), PDTC (3)	*BRAF* (6), WT (14)
Von Hinten et al. [31]	Retrospective	8	72 (65–87)	PTC (2), FTC (5)*, PDTC (1)	*BRAF* (1), *RAS* (1), WT (6)

Data are expressed as median (range) or mean ± standard deviation, as appropriate. *3 of these patients had oncocytic carcinoma. *FTC* follicular thyroid carcinoma; *NA* not available; *PDTC* poorly differentiated thyroid cancer; *PTC* papillary thyroid carcinoma; *WT* wild-type

**Table 2:** Characteristics of patients considered for the meta-analysis and therapeutic outcome measures

Study	Patients (n)	Drug (n)	Redifferentation method	Imaging modality	Outcome measures							
					Redifferentiation +				Redifferentiation −			
					CR	PR	SD	PD	CR	PR	SD	PD
Ho et al. [17]	20	Selumetinib (20)	PET/CT dosimetry	CT or MR	0	5	3	0	NA	NA	NA	NA
Rothenberg et al. [18]	10	Dabrafenib (10)	WBS interpretation	CT	0	2	4	0	0	0	2	1
Dunn et al. [19]	10	Vemurafenib (10)	PET/CT dosimetry	PET/CT	0	2	2	0	NA	NA	NA	NA
Tchekmedyian et al. [20]	6	Vemurafenib + anti-Erb3 antibody (6)	PET/CT dosimetry	CT or MR	0	2	2	0	NA	NA	NA	NA
Dotinga et al. [21]	9	Lenvatinib (9)	PET/CT dosimetry	PET/CT	NA	NA	NA	NA	0	4	3	2
Wadsley et al. [22]	9	Selumetinib (9)	WBS interpretation	SPECT/CT	NA	NA	NA	NA	NA	NA	NA	NA
Toro-Tobon et al. [23]	33	Dabrafenib + Trametinib (18), Trametinib (12), Other (3)	WBS interpretation	NA	0	2	13	2	0	0	8	3
Groussin et al. [24]	3	Larotrectinib (3)	WBS interpretation	PET/CT	0	0	0	2	0	0	0	1
Hoftijzer et al. [25]	21	Sorafenib (20)	WBS interpretation	SPECT/CT	NA	NA	NA	NA	NA	NA	NA	NA
Jaber et al. [26]	13	Dabrafenib (7), Trametinib (3), Other (3)	WBS interpretation	SPECT/CT	0	3	5	1	NA	NA	NA	NA
Leboulleux et al. [27]	10	Trametinib (10)	WBS interpretation	NA	NA	NA	NA	NA	NA	NA	NA	NA
Leboulleux et al. [28]	21	Dabrafenib + Trametinib (21)	WBS interpretation	NA	NA	NA	NA	NA	NA	NA	NA	NA
Pitoia et al. [29]	4	Dabrafenib (4)	WBS interpretation	SPECT/CT	0	1	2	0	0	0	1	0
Weber et al. [30]	20	Dabrafenib + Trametinib (20)	WBS interpretation	PET/CT	0	1	5	1	NA	NA	NA	NA
Von Hinten et al. [31]	8	Dabrafenib + Trametinib (8)	WBS interpretation	PET/CT	0	0	0	2	0	0	0	6

*CR* complete response; *PR* partial response; *SD* stable disease; *PD* progressive disease; *CT* computed tomography; *MR* magnetic resonance; *PET* positron emission tomography; *WBS* whole body scintigraphy; *SPECT* single photon emission computed tomography; *NA* not applicable/not available

### Assessment of the methodological quality of the included studies

**Fig. 2:**
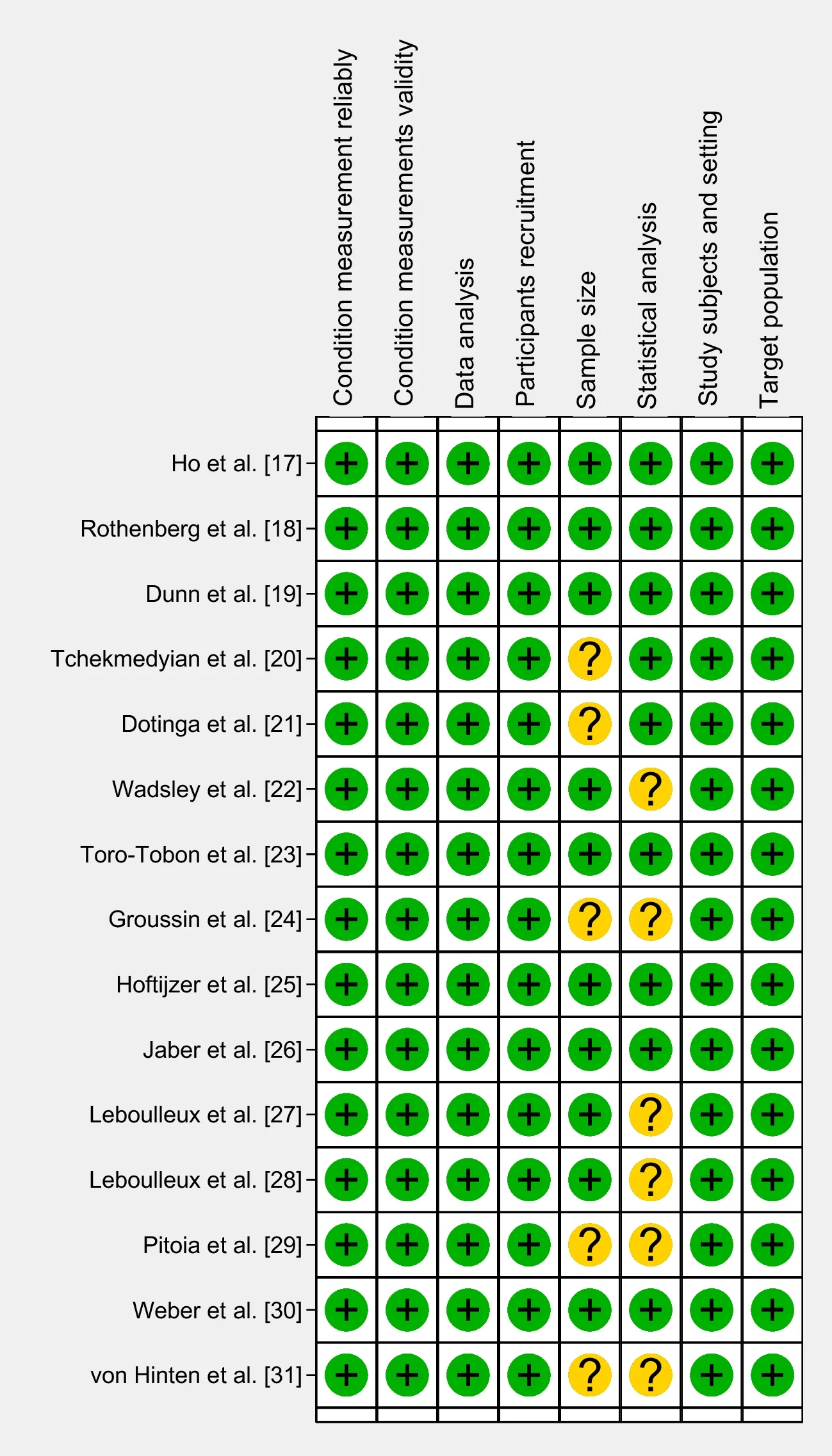
Methodological quality of the included studies assessed with the Joanna Briggs Institute Prevalence Critical Appraisal Tool. Green circles with a plus sign (+) indicate low risk of bias (criterion met), while yellow circles with a question mark (?) indicate an unclear risk of bias (insufficient information reported)

The quality assessment of the included studies is summarized in Fig. 2. Overall, the risk of bias was considered low in most studies. The domains that showed an unclear risk of bias were "sample size", likely attributable to an inadequate number of analyzed patients, which may compromise the precision of the final estimate, and "statistical analysis," specifically regarding the clear definition of the numerator and denominator.

### Pooled analysis of efficacy

**Fig. 3:**
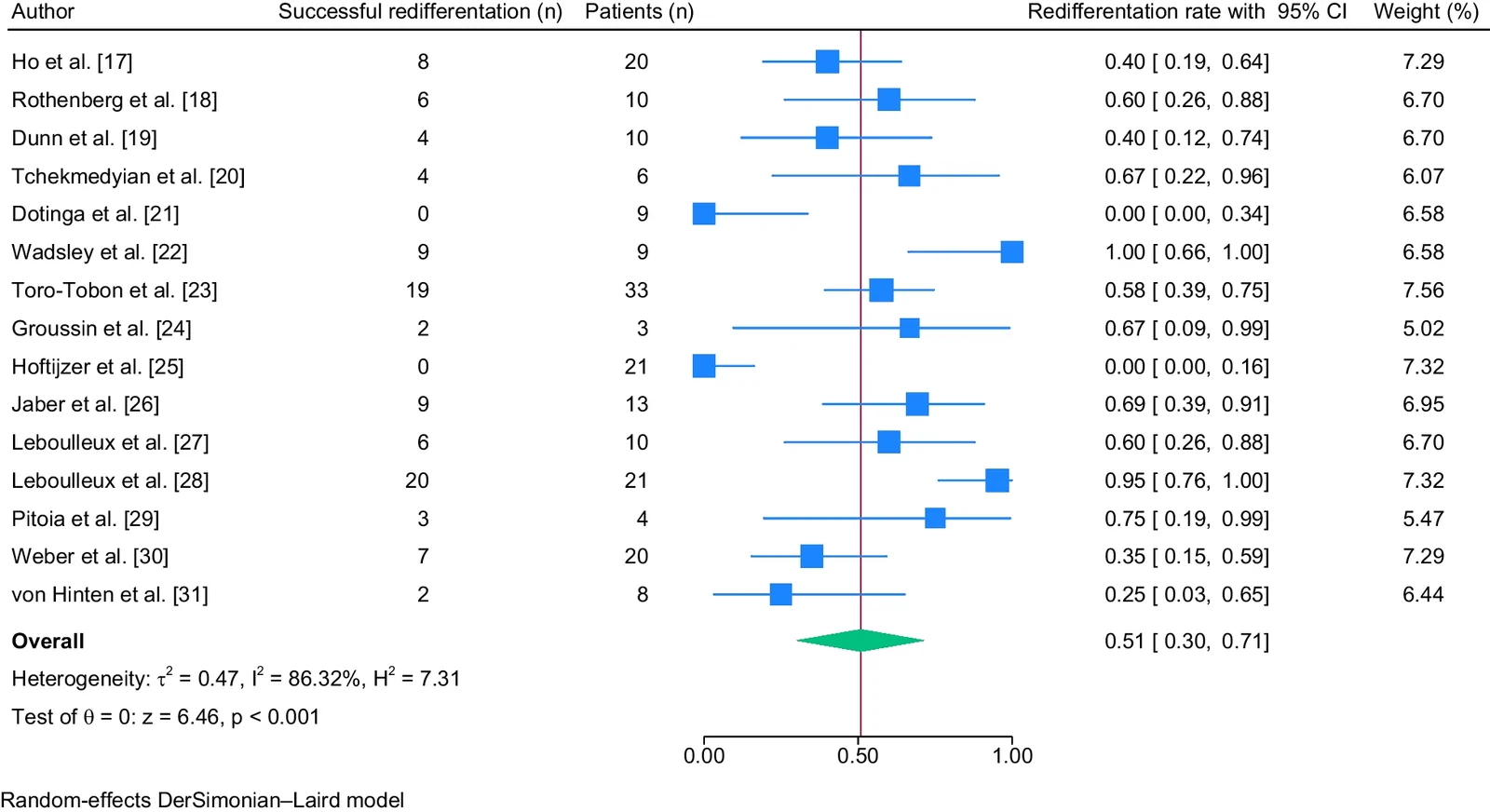
Forest plot of the overall successful redifferentiation rate. The horizontal lines represent the 95% confidence intervals of the point estimates. The area of each blue square is proportional to the study's weight in the pooled analysis. The green diamond represents the overall pooled estimate, with its lateral vertices indicating the 95% confidence interval. The vertical line represents the overall point estimate determined by the random-effects model. CI, confidence interval

**Fig. 4:**
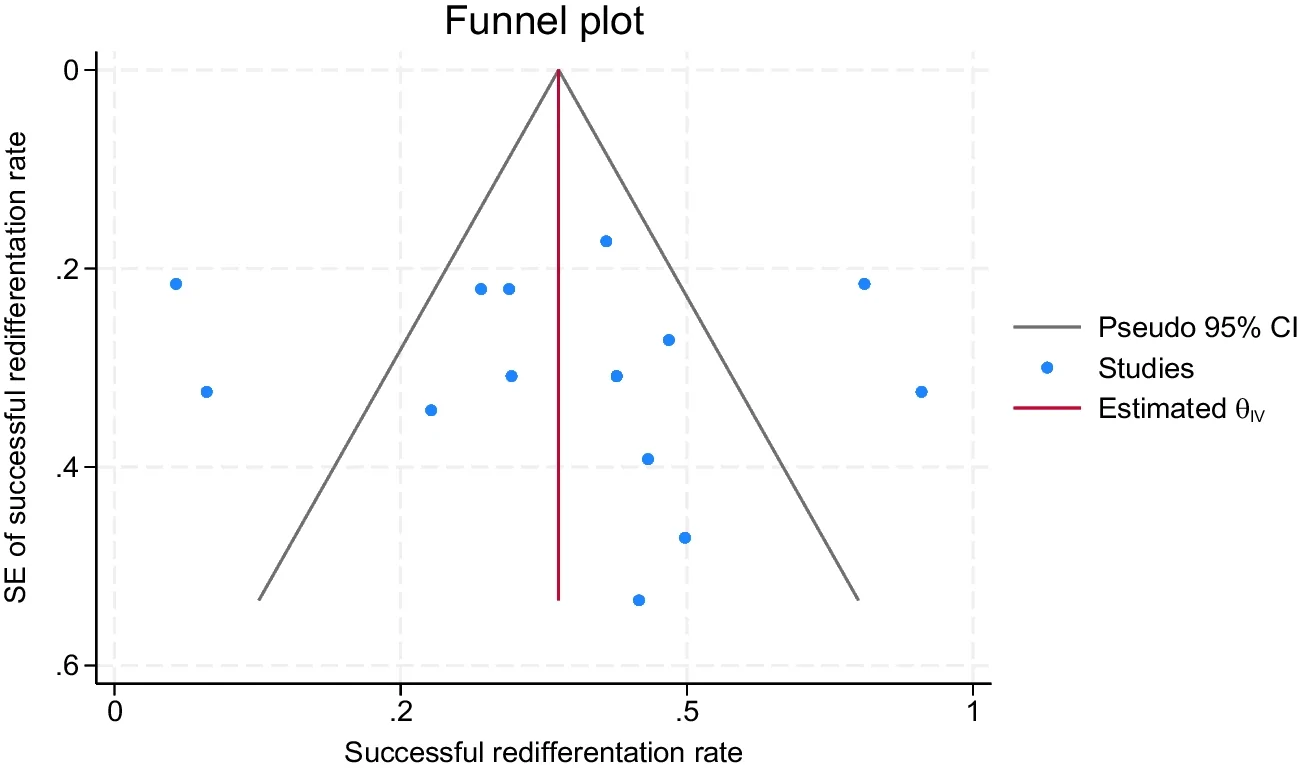
Funnel plot of the successful redifferentiation rate. Each blue dot represents an individual study; the y-axis represents study precision (standard error of the rate), and the x-axis shows the estimated effect size. Large studies appear toward the top of the graph and tend to cluster near the mean effect size, while small studies appear toward the bottom of the graph and are dispersed across a range of values due to higher sampling variation. The vertical red line represents the overall estimated effect, and the outer solid lines indicate the pseudo-95% confidence interval region within which studies are expected to lie in the absence of publication bias and heterogeneity. SE, standard error

The rate of successful redifferentiation ranged from 0% [21, 25] to 100%, [22] with a pooled estimated rate of 51% (95% confidence interval, CI: 30%−71%) (Fig. 3). A substantial and statistically significant inter-study heterogeneity was observed (*I*^*2*^ = 86%, τ^2^ = 0.47, *P* < 0.001), fully justifying the application of the random-effects DerSimonian-Laird model. The funnel plot indicated no significant publication bias among the included studies (*P* = 0.60) (Fig. 4). The leave-one-out sensitivity analysis demonstrated that no single study exerted a substantial influence on the overall effect size. At meta-regression analysis no significant association was found between the pooled estimate rate and study-level covariates analyzed.

### Pooled analysis of treatment response

**Fig. 5:**
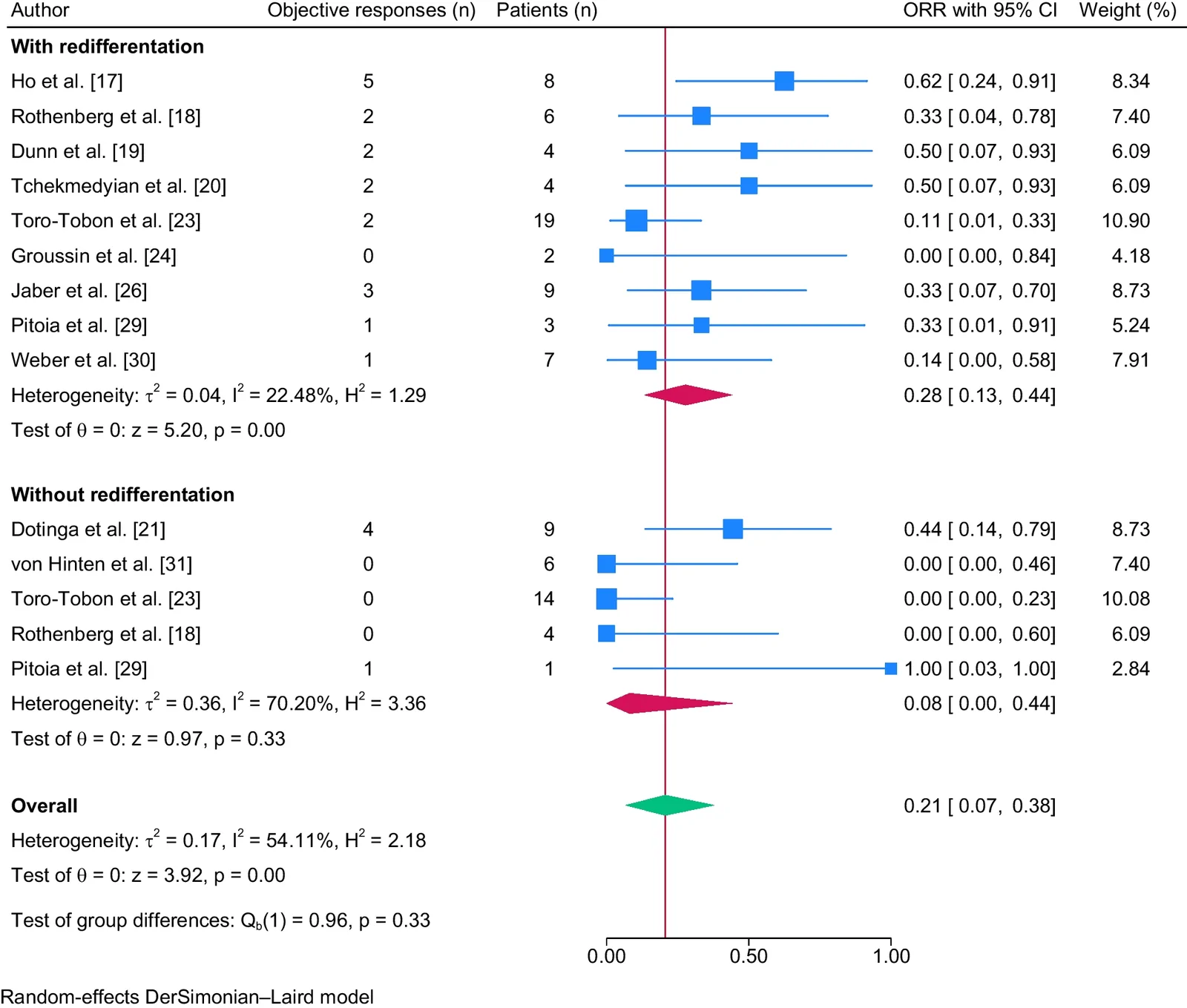
Forest plot of the objective response rate stratified by redifferentiation status. The horizontal lines represent the 95% confidence intervals of the point estimates for each study. The area of each blue square is proportional to the study's weight in the pooled analysis. The red diamonds represent the pooled estimates for each subgroup, while the green diamond represents the overall pooled estimate (the lateral vertices of the diamonds indicate the 95% confidence intervals). The vertical line represents the overall point estimate determined by the random-effects model. CI, confidence interval; ORR, objective response rate

**Fig. 6:**
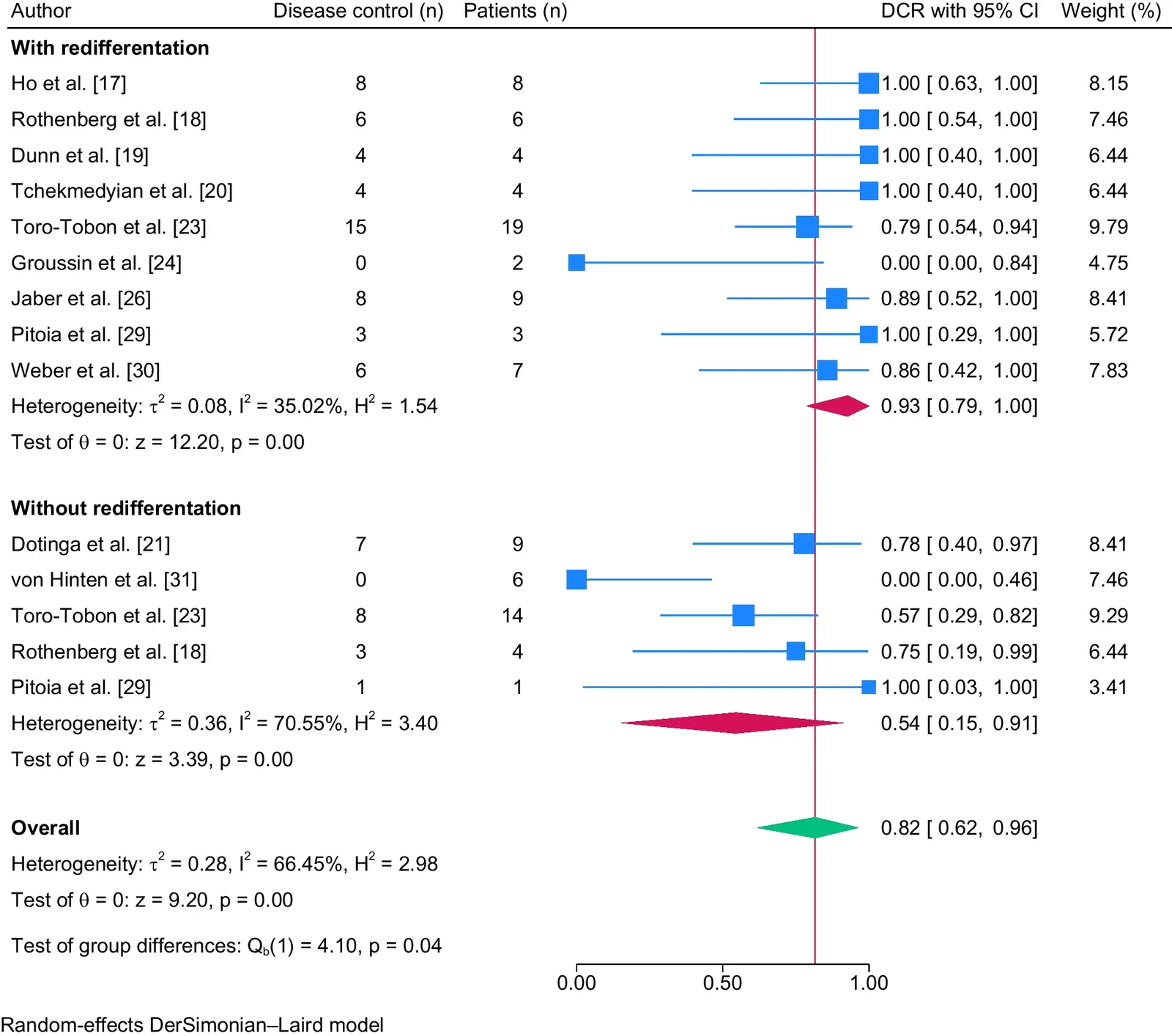
Forest plot of the disease control rate stratified by redifferentiation status. The horizontal lines represent the 95% confidence intervals of the point estimates for each study. The area of each blue square is proportional to the study's weight in the pooled analysis. The red diamonds represent the pooled estimates for each subgroup, while the green diamond represents the overall pooled estimate (the lateral vertices of the diamonds indicate the 95% confidence intervals). The vertical line represents the overall point estimate determined by the random-effects model. CI, confidence interval; DCR, disease control rate

Treatment response according to RECIST criteria was reported in 11 articles, encompassing a total of 93 patients (Table 2). The pooled ORR was 28% (95% CI: 13%−44%) in redifferentiated patients compared to 8% (95% CI: 0%−44%) in non-redifferentiated patients (Fig. 5). No statistically significant differences in ORR were observed between patients based on their redifferentiation status (*P* = 0.33). The pooled DCR was significantly higher, reaching 93% (95% CI: 79%−100%) in redifferentiated patients compared to 54% (95% CI: 15%−91%) in non-redifferentiated patients (*P* = 0.04) (Fig. 6).

### Subgroup analyses

Subgroup analyses based on the most likely sources of heterogeneity such as type of MAPK inhibitor and criteria for defining successful redifferentiation was also performed. No difference was identified in terms of type of MAPK inhibitor (*P* = 0.82) (Figure S1, Supplementary Material) and criteria for defining successful redifferentiation (*P* = 0.12) (Figure S2, Supplementary Material).

## Discussion

To our knowledge, this is the first meta-analysis exploring the efficacy of redifferentiation therapy in patients with RAI-refractory DTC. Administration of RAI is strongly recommended for the ablation of thyroid remnants in DTC patients at high risk of recurrence. Although the long-term prognosis for survival in DTC is generally excellent, clinical outcomes worsen significantly in the presence of distant metastases, being heavily influenced by factors such as patient age and tumor histology [32]. In metastatic settings, RAI therapy can improve survival primarily when the lesions exhibit high radioiodine avidity [4]. However, approximately two-thirds of patients with secondary lesions from DTC lose their capacity for radioiodine uptake due to dedifferentiation or intrinsic radio resistance [8]. According to the 2025 ATA guidelines, refractoriness to RAI treatment is defined as the absence of ^131^I uptake in known structural disease or as disease progression less than 6 months after RAI administration [3].

Genetic alterations causing constitutive activation of the MAPK pathway, including *BRAF* and *RAS* mutations, are strongly associated with the downregulated expression of proteins involved in iodine metabolism, a hallmark of most RAI-refractory thyroid cancers [6, 7, 33]. Consequently, redifferentiation therapy using MAPK inhibitors has been proposed to restore iodine uptake [8]. Once radioiodine avidity is successfully reinduced, a therapeutic dose of ^131^I can be administered. While previous studies have investigated the efficacy of MAPK inhibitors in restoring iodine concentration [17–30], with most being prospective trials [17–22, 25, 27, 28, 30], the present meta-analysis also integrates data on RAI-refractory from 5 retrospective case series [23, 24, 26, 29, 31] to provide a comprehensive overview.

The pioneering study in this field was conducted by Ho et al. [17] in 20 patients with RAI-refractory thyroid cancer treated with selumetinib to restore radioiodine uptake. The presence of redifferentiation was assessed via ^124^I positron emission tomography/computed tomography (PET/CT) dosimetry. A total of 8 patients reached the required dosimetry threshold and subsequently received ^131^I therapy; among these patients, response to therapy was assessed using RECIST criteria 6 months post-treatment, showing a PR in 5 patients and SD in 3. Similar encouraging results were observed by Wadsley et al. [22], where selumetinib administration successfully reinduced differentiation in all 9 patients evaluated.

Most published studies focus on patients harboring mutations in the MAPK pathway, particularly *BRAF* alterations, demonstrating consistent results in terms of redifferentiation rates across different targeted agents [17–20, 23, 26, 28–31]. Conversely, some authors observed no improvement in radioiodine uptake following the administration of lenvatinib [21] or sorafenib [25]. Our meta-analysis revealed a pooled redifferentiation rate of 51%, ranging from 0 to 100% [22]. These data indicate that approximately half of the patients treated with MAPK inhibitors achieve successful redifferentiation.

The highest rate of redifferentiation was reported by Leboulleux et al. [28] using a combination of dabrafenib and trametinib in 21 patients with *BRAF* V600 mutations. In their cohort, a total of 20 patients exhibited significant iodine uptake on post-therapeutic whole-body scan (WBS) after receiving a therapeutic dose of ^131^I, although most had not shown significant accumulation on diagnostic WBS scan.

Notably, redifferentiation rates vary among reports depending on the specific criteria used to define success, highlighting an urgent need for standardized definitions. The subjective interpretation of iodine uptake on post-therapy WBS can be confounding; therefore, most prospective studies define redifferentiation using a rigorous dosimetric strategy [17–20, 30]. This methodology is based on the quantification of the absorbed dose within metastatic lesions using different imaging modalities, including ^124^I PET/CT or diagnostic WBS. In those reports, patients with increased tracer uptake who failed to reach the predefined absorbed dose threshold were not considered to have achieved successful redifferentiation. The optimal absorbed dose threshold and the ideal imaging modality to define redifferentiation status remain to be fully elucidated.

In 11 studies, treatment response evaluated between 1 and 6 months after radioiodine therapy was also monitored. However, the fundamental question remains whether redifferentiation therapy is capable of improving long-term clinical outcomes. In our analysis, we compared ORR between patients with and without successful redifferentiation, revealing no statistically significant differences between the two groups. On the contrary, DCR was significantly higher in patients with successful redifferentiation compared to those without, although the wide CI in the non-redifferentiated group warrants cautious interpretation. While patients with restored radioiodine uptake generally proceeded to RAI therapy, non-redifferentiated patients usually followed alternative therapeutic pathways. Therefore, comparisons of clinical outcomes between these groups should be interpreted with caution.

Previous studies have demonstrated that in patients with distant metastases, the prognosis is significantly more favorable particularly among those with radioiodine-sensitive tumors [34, 35]. Whether the restoration of iodine uptake in poorly differentiated tumors exerts a significant impact on survival outcomes remains to be fully clarified. More recent studies evaluated the use of rearranged during transfection (RET) inhibitors in redifferentiation therapy to restore iodine uptake in metastatic lesions of iodine-refractory thyroid cancer [36, 37]. These drugs have shown potential redifferentiation activity in RAI-refractory differentiated thyroid cancer with RET rearrangements/fusions. The sustained efficacy and good tolerability of these drugs underscore the importance of identifying the optimal time to evaluate redifferentiation and plan subsequent radiometabolic therapy, as part of a combination strategy that could, in selected cases, allow temporary or permanent interruption of systemic treatment [34]. Consequently, more consistent data from larger patient populations are urgently needed to definitively assess the potential impact of this therapeutic strategy on patient outcomes. To date, a statistically significant improvement in progression-free survival and overall survival in redifferentiated patients subsequently treated with RAI has not yet been demonstrated.

Several limitations must be acknowledged when interpreting the findings of this meta-analysis. First, the total sample size of the pooled population remains relatively small (n = 234), reflecting the rarity of the clinical condition and the experimental nature of redifferentiation protocols. Second, there was a substantial degree of study-level heterogeneity, particularly concerning the type and dosage of targeted therapies administered (e.g., MEK inhibitors versus *BRAF* inhibitors or multi-kinase inhibitors) and the specific histological subtypes included across the cohorts. Third, the criteria used to define a successful "redifferentiation status" were not uniform across the selected literature. While most prospective trials relied on a rigorous, quantitative ^124^I PET/CT dosimetric approach, several retrospective series evaluated re-uptake based on the qualitative or subjective interpretation of diagnostic or post-therapy ^131^I WBS. Lastly, the follow-up period in the majority of the included studies was limited to short-term tumor response assessments (1 to 6 months), which prevents a definitive evaluation of the true impact of redifferentiation therapy on robust, long-term survival endpoints such as progression-free survival and overall survival.

## Conclusions

In patients with RAI-refractory thyroid tumors, redifferentiation therapy using MAPK inhibitors is capable of restoring radioiodine avidity in approximately half of the pooled population. Although short-term disease control rates are promising, statistically significant prognostic differences and long-term survival benefits remain to be demonstrated in larger, standardized trials with extended follow-up.

## Supplementary Information

Below is the link to the electronic supplementary material.

Supplementary file1 Forest plot of successful redifferentiation rate by mitogen-activated protein kinase (MAPK) inhibitor class. The horizontal lines represent the 95% confidence intervals of the point estimates. The area of each blue square is proportional to the study's weight in the pooled analysis. The red diamonds represent the pooled estimates for each inhibitor subgroup, while the green diamond represents the overall pooled estimate (the lateral vertices of the diamonds indicate the 95% confidence intervals). The vertical line represents the overall point estimate determined by the random-effects model. BRAF, Raf murine sarcoma viral oncogene homolog B; CI, confidence interval; MAPK, mitogen-activated protein kinase; MEK, mitogen-activated protein kinase kinase. (PDF 126 KB)

Supplementary file2 Forest plot of successful redifferentiation rate according to the criteria used for defining successful redifferentiation. The horizontal lines represent the 95% confidence intervals of the point estimates. The area of each blue square is proportional to the study's weight in the pooled analysis. The red diamonds represent the pooled estimates for each diagnostic criteria subgroup, while the green diamond represents the overall pooled estimate (the lateral vertices of the diamonds indicate the 95% confidence intervals). The vertical line represents the overall point estimate determined by the random-effects model. CI, confidence interval; PET/CT, positron emission tomography/computed tomography; WBS, whole-body scintigraphy. (PDF 126 KB)

## Data Availability

All data generated or analyzed during this study are included in this published article and its supplementary information files.
