# Supplementary material for: Efficacy of redifferentiation therapy in radioactive iodine refractory thyroid cancer: a systematic review and meta-analysis
Source: Eur J Nucl Med Mol Imaging. 2026 Aug 6;53(13):8177–88. doi: 10.1007/s00259-026-08115-7 (PMC13727798; doi:10.1007/s00259-026-08115-7)
Supplement: Supplementary file 1 [file 259_2026_8115_MOESM1_ESM.pdf]

Author	Successful redifferentation (n)	Patients (n)	Redifferentation rate with 95% CI	Weight (%)
**BRAF inhibitor**				
Rothenberg et al. [18]	6	10	0.60 [0.26, 0.88]	6.70
Dunn et al. [19]	4	10	0.40 [0.12, 0.74]	6.70
Tchekmedyian et al. [20]	4	6	0.67 [0.22, 0.96]	6.07
Pitoia et al. [29]	3	4	0.75 [0.19, 0.99]	5.47
Heterogeneity: $\tau^2 = 0.00$, $I^2 = 0.00\%$, $H^2 = 1.00$			0.57 [0.37, 0.76]	
Test of $\theta = 0$: z = 7.44, p = 0.00				
**MEK inhibitor**				
Leboulleux et al. [27]	6	10	0.60 [0.26, 0.88]	6.70
Heterogeneity: $\tau^2 = 0.00$, $I^2 = .\%$, $H^2 = .$			0.60 [0.28, 0.89]	
Test of $\theta = 0$: z = 4.69, p = 0.00				
**Multi-kinase inhibitor**				
Ho et al. [17]	8	20	0.40 [0.19, 0.64]	7.29
Dotinga et al. [21]	0	9	0.00 [0.00, 0.34]	6.58
Wadsley et al. [22]	9	9	1.00 [0.66, 1.00]	6.58
Toro-Tobon et al. [23]	19	33	0.58 [0.39, 0.75]	7.56
Groussin et al. [24]	2	3	0.67 [0.09, 0.99]	5.02
Hoftijzer et al. [25]	0	21	0.00 [0.00, 0.16]	7.32
Jaber et al. [26]	9	13	0.69 [0.39, 0.91]	6.95
Leboulleux et al. [28]	20	21	0.95 [0.76, 1.00]	7.32
Weber et al. [30]	7	20	0.35 [0.15, 0.59]	7.29
von Hinten et al. [31]	2	8	0.25 [0.03, 0.65]	6.44
Heterogeneity: $\tau^2 = 0.64$, $I^2 = 90.94\%$, $H^2 = 11.04$			0.47 [0.20, 0.75]	
Test of $\theta = 0$: z = 4.51, p = 0.00				
**Overall**			0.51 [0.30, 0.71]	
Heterogeneity: $\tau^2 = 0.47$, $I^2 = 86.32\%$, $H^2 = 7.31$				
Test of $\theta = 0$: z = 6.46, p = 0.00				
Test of group differences: $Q_b(2) = 0.40$, p = 0.82				

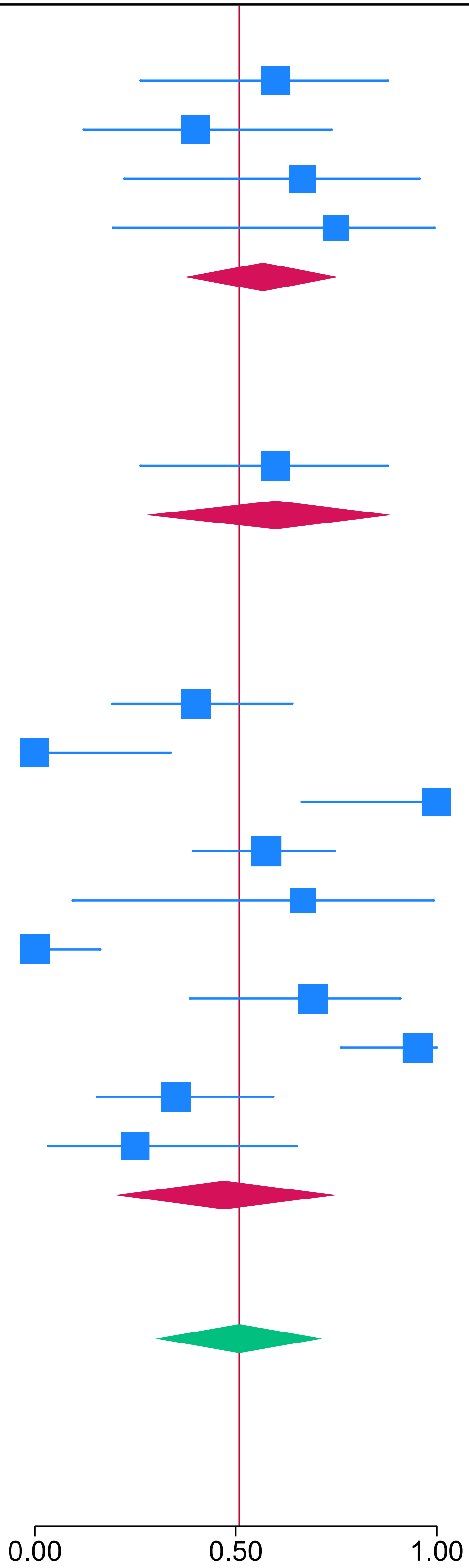

Random-effects DerSimonian–Laird model
