# Supplementary material for: Efficacy of redifferentiation therapy in radioactive iodine refractory thyroid cancer: a systematic review and meta-analysis
Source: Eur J Nucl Med Mol Imaging. 2026 Aug 6;53(13):8177–88. doi: 10.1007/s00259-026-08115-7 (PMC13727798; doi:10.1007/s00259-026-08115-7)
Supplement: Supplementary file 2 [file 259_2026_8115_MOESM2_ESM.pdf]

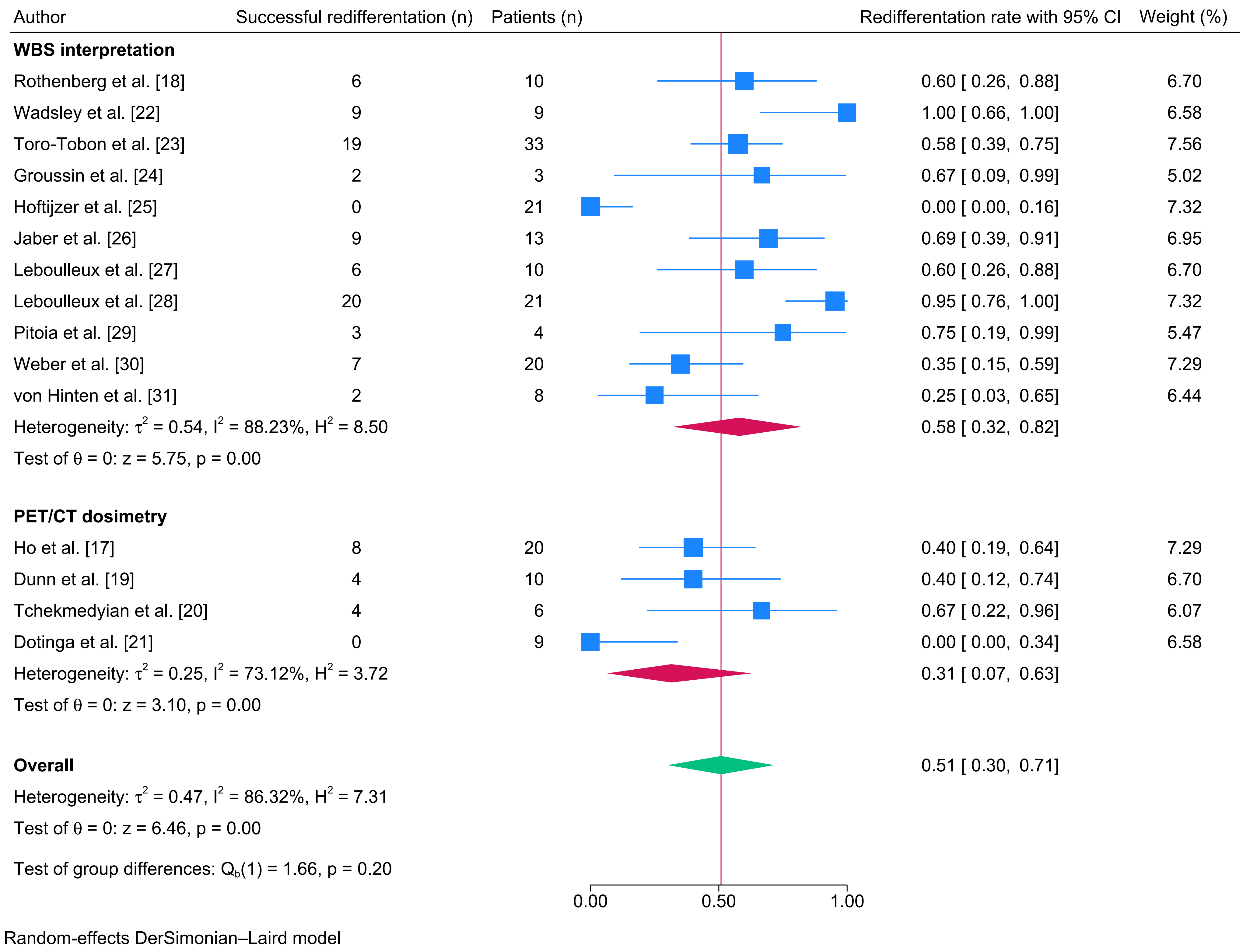

Author
Successful redifferentation (n)
Patients (n)
Redifferentation rate with 95% CI
Weight (%)
WBS interpretation
Rothenberg et al. [18] 6 10 0.60 [0.26, 0.88] 6.70
Wadsley et al. [22] 9 9 1.00 [0.66, 1.00] 6.58
Toro-Tobon et al. [23] 19 33 0.58 [0.39, 0.75] 7.56
Groussin et al. [24] 2 3 0.67 [0.09, 0.99] 5.02
Hoftijzer et al. [25] 0 21 0.00 [0.00, 0.16] 7.32
Jaber et al. [26] 9 13 0.69 [0.39, 0.91] 6.95
Leboulleux et al. [27] 6 10 0.60 [0.26, 0.88] 6.70
Leboulleux et al. [28] 20 21 0.95 [0.76, 1.00] 7.32
Pitoia et al. [29] 3 4 0.75 [0.19, 0.99] 5.47
Weber et al. [30] 7 20 0.35 [0.15, 0.59] 7.29
von Hinten et al. [31] 2 8 0.25 [0.03, 0.65] 6.44
Heterogeneity: τ^2 = 0.54, I^2 = 88.23%, H^2 = 8.50 0.58 [0.32, 0.82]
Test of θ = 0: z = 5.75, p = 0.00
PET/CT dosimetry
Ho et al. [17] 8 20 0.40 [0.19, 0.64] 7.29
Dunn et al. [19] 4 10 0.40 [0.12, 0.74] 6.70
Tchekmedyian et al. [20] 4 6 0.67 [0.22, 0.96] 6.07
Dotinga et al. [21] 0 9 0.00 [0.00, 0.34] 6.58
Heterogeneity: τ^2 = 0.25, I^2 = 73.12%, H^2 = 3.72 0.31 [0.07, 0.63]
Test of θ = 0: z = 3.10, p = 0.00
Overall 0.51 [0.30, 0.71]
Heterogeneity: τ^2 = 0.47, I^2 = 86.32%, H^2 = 7.31
Test of θ = 0: z = 6.46, p = 0.00
Test of group differences: $Q_b(1)$ = 1.66, p = 0.20
0.00
0.50
1.00
Random-effects DerSimonian–Laird model
